# Nasal tissues from Alzheimer’s disease patients induces the formation of aggregates in a cellular model of tau propagation

**DOI:** 10.1002/alz.084341

**Published:** 2025-01-09

**Authors:** Daijiro Yanagisawa, Aslina Pahrudin Arrozi, Tomoko Kato, Tamio Mizukami, Hiroyasu Akatsu, Hashizume Yoshio, Daita Kaneda, Ikuo Tooyama

**Affiliations:** ^1^ Shiga University of Medical Science, Otsu Japan; ^2^ University of Malaya, Kuala Lumpur Malaysia; ^3^ Nagahama Institute of Bio‐Science and Technology, Nagahama Japan; ^4^ Nagoya City University Graduate School of Medical Sciences, Nagoya, Aichi Japan; ^5^ Institute of Neuropathology, Fukushimura Hospital, Toyohashi Japan

## Abstract

**Background:**

Abnormal protein depositions of amyloid β and tau are present in the nasal cavity in patients with Alzheimer’s disease. This finding raises an idea that nasal tissues would be a promising source of diagnostic biomarkers for Alzheimer’s disease. However, the amounts of amyloid β and tau are extremely small, making it difficult to quantify the levels using conventional methods such as ELISA, and thus it is challenging to utilize them for the diagnostic biomarkers. In the present study, we tested the nasal tissues as the source of diagnostic biomarkers using a cellular model of tau propagation.

**Method:**

This study was approved by the institutional Ethics Committees. Postmortem nasal mucosa and brain tissues from patients with AD (n = 10) and normal subjects (n = 10) were collected with patient consent at the Fukushimura Brain Bank. Nasal and brain tissue homogenates were added to HEK293 cells expressing tau 3‐repeat domain with the L266V and V337M mutations (3RD^∗^VM) or 4‐repeat domain with the P301L and V337M mutations (4RD^∗^LM), which was fused with GFP at the C‐terminus.

**Result:**

GFP fluorescence was detected uniformly within the cell bodies of HEK293T cells expressing 3RD^∗^VM‐EGFP and 4RD^∗^LM‐EGFP. There were no changes in the fluorescence after the additions of the brain homogenates from normal subjects. In contrast, a large number of fluorescent puncta was detected both in HEK293T cells expressing 3RD^∗^VM‐EGFP and 4RD^∗^LM‐EGFP at 4 days after the additions of the brain homogenates from patients with AD. Furthermore, the nasal tissue homogenates from patients with AD also induce the formation of fluorescent aggregates in HEK293T cells expressing 3RD^∗^VM‐EGFP and 4RD^∗^LM‐EGFP. Quantitative analysis revealed that the nasal tissue homogenates from AD patients significantly induced the aggregate formation, compared with normal subjects.

**Conclusion:**

These results suggest that the nasal tissues from AD patients contain tau seeds with prion activity, similar to the brain. A cellular bioassay using nasal tissues would be great potential as an AD biomarker because of the usefulness of nasal tissue biopsy, and would provide an important contribution to the development of ex vivo diagnosis method for AD using the nasal extracts.

